# Surgical resolution of chronic thoracic pain stemming from a rare osteo-muscular conflict: A case report

**DOI:** 10.1016/j.ijscr.2024.109589

**Published:** 2024-03-28

**Authors:** Fabio Vita, Fabio Davoli, Galletti Stefano, Riccardo Ferri, Roberto Tedeschi, Danilo Donati

**Affiliations:** a IRCCS Istituto Ortopedico Rizzoli, Orthopaedics and Traumatology Clinic, University of Bologna, Bologna, Italy; b Department of Biomedical and Neuromotor Sciences, Alma Mater Studiorum, University of Bologna, Bologna, Italy; c University of Modena and Reggio Emilia, Largo del Pozzo 71, 41124 Modena, Italy; d General & Thoracic Surgery Unit, IRCCS Istituto Ortopedico Rizzoli, Bologna, Italy

**Keywords:** Chronic thoracic pain, Musculoskeletal disorders, Rib-muscle conflict, Surgical intervention, Personalized medicine in orthopedics

## Abstract

**Introduction:**

Chronic thoracic pain presents significant diagnostic and therapeutic challenges, particularly when arising from rare osteo-muscular conflicts. This report details a unique case of chronic pain due to an osteo-muscular conflict between the right tenth rib and the internal oblique muscle, highlighting the complexities involved in diagnosis and the potential for surgical resolution.

**Case presentation:**

A 33-year-old male with a decade-long history of chronic right hemithorax pain, unresponsive to conservative treatments, underwent diagnostic evaluation. Advanced imaging techniques, including a thoracic CT scan, revealed an ipodense area between the ninth and tenth ribs, suggesting an osteo-muscular conflict. Surgical intervention, specifically a partial costectomy of the right tenth rib, was pursued, resulting in significant symptom relief and improved quality of life.

**Clinical discussion:**

This case underscores the importance of considering advanced diagnostic evaluations in persistent chronic pain cases and the effectiveness of targeted surgical interventions in resolving anatomical conflicts. It contributes to the body of knowledge on managing complex musculoskeletal conditions and underscores the need for personalized treatment approaches.

**Conclusion:**

Surgical intervention in selected cases of chronic pain due to rare anatomical conflicts can offer significant relief and enhance patient outcomes. This case advocates for a nuanced approach to the diagnosis and treatment of chronic thoracic pain, emphasizing the role of advanced imaging and the potential benefits of surgical resolution.

## Highlights

• Rare rib-muscle conflict resolved surgically.
• Advanced imaging uncovers chronic pain cause.
• Partial costectomy significantly improves life.
• Case prompts reevaluation of chronic pain.
• Insights on treating musculoskeletal conditions.

## 1 Introduction

The Slipping rib syndrome (SRS), also known as clicking rib, is a lesser-known musculoskeletal condition that manifests as a slipping or clicking sensation in the lower thoracic region or upper abdomen [1]. It is considered a rare syndrome, accounting for approximately 5 % of all musculoskeletal chest pain encountered in primary care settings [2]. The syndrome can manifest at any age but is more commonly observed in middle-aged women and is a recognized, though relatively rare, cause of recurrent lower thoracic and/or upper abdominal pain in adolescents [3,4]. Cases have been reported in individuals ranging from 7 to 86 years old, with a higher prevalence in the middle-aged population.

This phenomenon can occur during specific torso movements, such as bending, twisting, or lifting weights [5]. While some individuals may experience this condition without pain, others may suffer significant discomfort or pain [6,7]. Etiologically, slipping rib syndrome can arise from various causes, including abnormal movements of one or more ribs, which may be due to muscle weakness, previous injuries, or anatomical anomalies [8,9]. Another potential cause is the interference between the rib and surrounding tissue, such as muscles or ligaments, which can produce a snapping sensation as the rib moves in certain ways. Additionally, inflammation of the costal joint structures, like the costovertebral or costosternal joints, can alter the normal movement of the ribs, contributing to the syndrome [[10], [11], [12]].

**Fig. 1:**
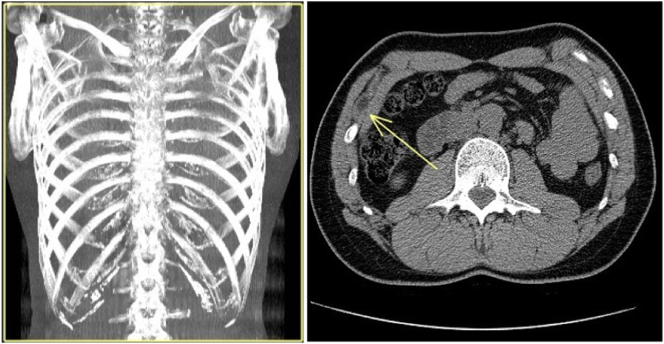
Computed Tomography. “Composite CT scan showcasing anatomical details of the thoracic region, emphasizing the area of osteo-muscular conflict.”

Many patients may not seek medical attention for mild symptoms or may be misdiagnosed with other thoracic conditions [[12], [13], [14]]. Treatment varies based on the severity of symptoms and the underlying cause and can include a combination of rest, modifications to daily activities to avoid movements that provoke the snapping, physical therapy to strengthen the thoracic muscles and improve mobility, the application of heat or ice to manage pain and inflammation, and the use of non-steroidal anti-inflammatory drugs (NSAIDs) to reduce pain and inflammation [[15], [16], [17]]. In more severe or persistent cases, corticosteroid injections or, rarely, surgical interventions may be considered. In the context of slipping rib syndrome, when conservative treatments fail to provide significant relief or when the condition becomes particularly debilitating, surgical options may be considered [18]. Although rare, the surgical approach proves to be a definitive solution for selected cases, aiming to eliminate the source of pain and physical slipping through the removal of the involved rib portion or correction of the surrounding structures contributing to the issue [19]. The decision to proceed with surgery requires careful evaluation by a multidisciplinary team [20], including thoracic surgeons, orthopedic specialists, and physical therapists, to ensure that the benefits outweigh the potential risks. Preoperative planning might involve advanced imaging studies, such as magnetic resonance imaging (MRI) or computed tomography (CT) (Fig. 1), to precisely map the patient's thoracic anatomy and accurately identify the location and nature of the anomalies involved. Surgical intervention for slipping rib syndrome can range from minimally invasive to more extensive, depending on the case's specificity. Minimally invasive techniques, such as video-assisted thoracoscopic surgery (VATS), reduce surgical trauma and shorten recovery times, offering the patient a quicker and less painful recovery. These techniques allow the surgeon to view the inside of the chest through small incisions, using specialized instruments to remove or correct the involved rib or structures. The purpose of this case report is to provide a detailed examination of a patient with slipping rib syndrome, aiming to augment the current understanding of its presentation, management, and outcomes. Through this report, we intend to shed light on the diagnostic challenges and therapeutic approaches, potentially contributing to a more systematic recognition and treatment of this underrecognized condition in clinical practice.

## 2 Case presentation

The patient, a 33-year-old male who had endured a decade of persistent, chronic pain localized to the right hemithorax, sought treatment at our hospital, which specializes in orthopedics. His journey to find relief for his symptoms included consultations with primary care physicians and various specialists, including physiotherapists, osteopaths, and chiropractors. Despite these consultations, no significant underlying illness was identified, and the treatments, varying from myofascial dysfunction management to fibromyalgia hypotheses, were conservative, focusing on pain alleviation without invasive interventions. No specific blood tests were conducted for this condition, except for standard pre-surgical anesthetic evaluations, which showed no abnormal hematological parameters.

Given the localized nature of the pain and the ineffectiveness of conservative treatments, a thoracic CT scan was performed as part of our diagnostic process, revealing a hypodense segment between the ninth and tenth ribs. This finding raised suspicions of an osteoarticular issue, leading to the decision against an MRI. While MRI could theoretically provide more detailed soft tissue images, it was not expected to offer additional diagnostic clarity for this specific osteo-muscular concern.

The surgical approach focused on addressing the identified osteo-muscular conflict, and the excised segment was not sent for histopathological examination. This decision was based on the benign nature of the pathology and the clear diagnostic findings from imaging studies, which sufficiently explained the patient's symptoms without the need for further histological analysis.

Our involvement in the patient's care was as specialists in an orthopedic hospital, where he was referred after years of unsuccessful treatment attempts. Our role was to offer a surgical solution to a problem that had been approached non-invasively yet conservatively by other professionals, focusing on a diagnosis that pointed to an anatomical conflict as the source of his chronic pain.

## 3 Clinical findings

### 3.1 Physical examination

The physical examination revealed several key findings:

• Tenderness: Marked tenderness was noted upon palpation of the right lateral chest wall, particularly in the area between the ninth and tenth ribs.
• Pain Provocation: Certain movements, such as deep inhalation, coughing, and twisting of the torso, elicited sharp pain radiating from the tender area towards the epigastric region. The intensity of the pain was evaluated using the Numeric Pain Rating Scale (NPRS), where it was rated as 6/10, indicating a moderate level of discomfort.
• Audible/Perceptible Phenomena: No audible snaps, clicks, or crepitus were detected during the examination, which could be indicative of snapping rib syndrome but were absent in this case.
• Neurovascular Examination: The neurovascular examination of the upper limbs and thoracic region was unremarkable, with no signs of neurological deficit or vascular compromise.

### 3.2 Diagnostic imaging

Given the chronic nature of the pain and the specific findings from the physical examination, further diagnostic imaging was pursued to elucidate the underlying cause:

• Thoracic Computed Tomography (CT) Scan: Performed on June 14, 2023, the CT scan revealed an ipodense area measuring approximately 1.5 cm in axial diameter situated between the ninth and tenth ribs near their costal junctions. This finding suggested the presence of potential lacerative muscular insertion outcomes, likely involving the proximal junction of the internal oblique muscle. Additionally, a partially calcific appearance of the costosternal joints was observed on both sides, which could contribute to the patient's symptomatology through altered biomechanical dynamics.

These clinical findings, characterized by localized pain exacerbated by movement and specific physical examination results, prompted further investigation into the structural and anatomical aspects of the patient's condition. The diagnostic imaging provided crucial insights into the anatomical anomalies contributing to the patient's symptoms, guiding the decision-making process for therapeutic intervention. This case study adheres to the SCARE (Surgical Case Report) guidelines for reporting surgical case studies [21,22].

### 3.3 Timeline

**Table:** 

Date	Event
2003–2013	Patient experiences intermittent to chronic pain in the right hemithorax without seeking medical attention.
June 14, 2023	Thoracic CT scan reveals an ipodense area between the ninth and tenth ribs, suggesting potential lacerative muscular insertion outcomes, and partial calcification of the costosternal joints.
June–August 2023	Patient undergoes evaluation, including physical therapy and conservative management, which do not yield significant relief.
September 4, 2023	Pre-operative ultrasound marking performed, targeting the right tenth rib for surgical intervention.
September 5, 2023	Partial costectomy of the right tenth rib is performed to address the osteo-muscular conflict and chronic pain.
September–October 2023	Postoperative recovery shows significant pain improvement and enhanced quality of life. The patient begins a tailored physical rehabilitation program.
December 2023	Follow-up assessment indicates sustained symptom improvement; the patient reports reduced pain and increased daily activity engagement.
February 2024	Stable symptom improvement with no recurrence; further follow-ups planned to monitor long-term outcomes.

This table offers a quick reference to the chronological progression of the patient's case, from the initial symptom onset through diagnostic processes, intervention, and recovery, highlighting the effectiveness of the surgical treatment in alleviating the patient's chronic pain.

### 3.4 Diagnostic assessment

During the initial medical assessment, a thorough physical examination was conducted, revealing sensitivity and eliciting sharp pain upon palpation of the right side chest wall, with the discomfort extending towards the epigastric area. The persistent nature of the pain, its precise location, and the lack of relief from standard pain relief methods underscored the need for a more detailed diagnostic exploration.

On June 14, 2023, a thoracic CT scan was undertaken, uncovering a notable finding: a hypodense region about 1.5 cm in diameter located at the intersection of the ninth and tenth ribs. This finding indicated potential lacerative changes at the muscle's insertion point, particularly at the proximal junction of the internal oblique muscle. The imaging also displayed a calcific pattern within the costosternal joints on both sides. While this may be coincidental, it's plausible that such changes could influence the patient's pain through shifts in the biomechanical balance.

### 3.5 Therapeutic intervention

**Fig. 2:**
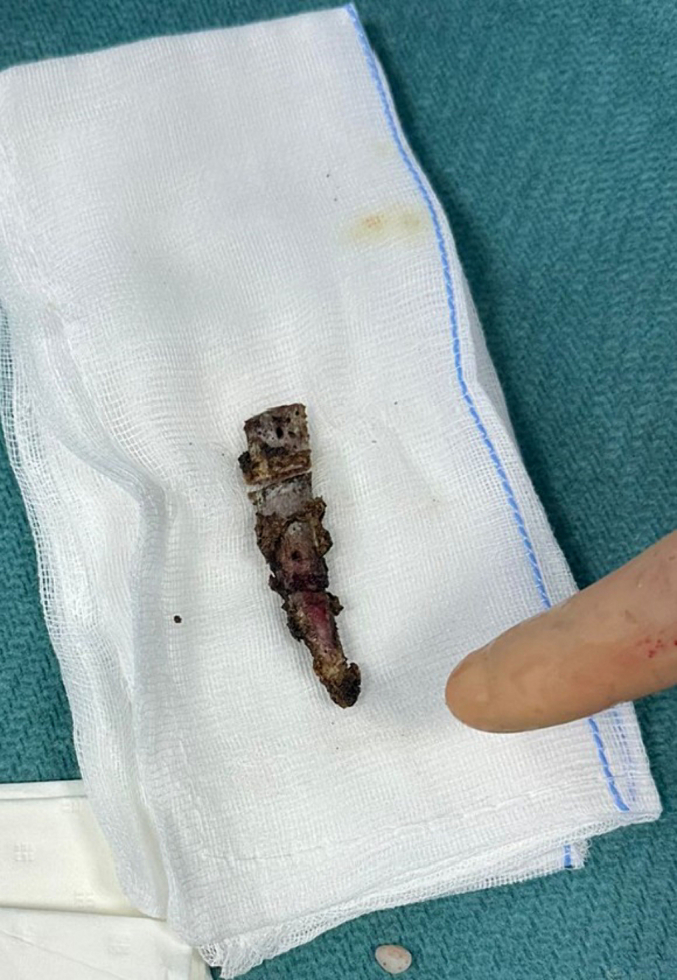
Resected supernumerary rib: post-surgical specimen. “Resected tissue specimen showing the removed segment of a rib affected by slipping rib syndrome, following surgical intervention aimed at alleviating thoracic pain.”

After enduring chronic pain for ten years without a clear diagnosis, the patient finally received a definitive explanation for their suffering through an advanced thoracic CT scan, which revealed an osteo-muscular conflict. With this newfound clarity and the prospect of a surgical solution that promised significant relief, the patient, deeply affected by a decade of unresolved pain, was strongly inclined towards the surgical option. This mutual decision between the patient and the medical team was driven by the prolonged duration of suffering, the ineffectiveness of conservative treatments, and a shared conviction in the potential of surgery to provide the long-awaited resolution. On September 5, 2023, a partial costectomy of the right tenth rib was performed (Fig. 2). This procedure was meticulously planned, with pre-operative ultrasound marking conducted on September 4, 2023, to ensure precise identification and removal of the problematic rib segment. The chosen surgical intervention for the patient, a partial costectomy, was meticulously designed to alleviate the chronic pain caused by the conflict between the right tenth rib and the internal oblique muscle. This procedure entailed the precise removal of the problematic rib segment under general anesthesia. A carefully planned incision allowed for minimal invasiveness, with the surgical team using advanced imaging to guide the resection of the affected rib and any involved muscular attachments [23,24]. The goal was to eliminate the source of pain while maintaining thoracic integrity. Post-operative care focused on pain management, respiratory exercises, and, if necessary, physical therapy [25,26] to ensure a swift recovery and restoration of full function. This tailored approach was selected after exhaustive exploration of non-surgical options, aiming to significantly improve the patient's quality of life.

### 3.6 Follow-up and outcomes

The surgical intervention marked a turning point in the patient's condition. Postoperatively, the patient experienced a dramatic reduction in pain levels, with subsequent follow-ups indicating a sustained improvement in his quality of life. The success of the surgery underscored the importance of accurately diagnosing and addressing the underlying anatomical abnormalities contributing to chronic pain syndromes.

## 4 Discussion

In the compelling case of a 33-year-old male enduring chronic pain due to an osteo-muscular conflict between the right tenth rib and the internal oblique muscle, the intricacies of musculoskeletal medicine are brought to the forefront. This scenario illuminates the profound challenges encountered in both diagnosing and managing such uncommon conditions, further complicated by a decade of ineffective conservative treatment approaches, yet ultimately resolved through a strategic surgical intervention [6,12].

The discussion commences by underscoring the uniqueness of this case, highlighting its importance due to the rare nature of the osteo-muscular conflict and its significant contribution to the patient's prolonged suffering. The case's novelty lies in its addressal of a scarcely explored cause of chronic thoracic pain, thereby bridging a critical knowledge gap within the domain of musculoskeletal disorders [1,5].

A meticulous review of pertinent literature situates this case within a broader scientific context, emphasizing the indispensable role of advanced imaging techniques. Specifically, thoracic CT scans have proven pivotal in identifying the anatomical anomalies that underpin chronic pain conditions, marking a significant contribution to the musculoskeletal diagnostic process [10,27,28]. By drawing parallels with similar instances of rib-muscle conflict, this case broadens the recognized spectrum of etiologies for chronic thoracic pain, enhancing the medical community's understanding and management strategies.

The narrative then delves into the diagnostic and therapeutic conundrums presented by this case. The absence of overt physical signs and the subsequent reliance on sophisticated diagnostic tools underscore the complexities involved in reaching a definitive diagnosis. Furthermore, the deliberation leading to the surgical resolution aligns with existing research that advocates for operative solutions in instances where identifiable anatomical causes of chronic pain are present, thus emphasizing the necessity for personalized surgical planning and pre-operative imaging for accurate intervention [6,12].

This case compels a reevaluation of the clinical approach to patients presenting with non-specific chronic thoracic pain. It suggests that a more assertive diagnostic workup, inclusive of advanced imaging, may be warranted in persistent cases unresponsive to conservative treatment modalities. Moreover, it showcases the potential advantages of surgical intervention as a component of the treatment algorithm for analogous clinical scenarios, thereby advocating for its consideration [1,5].

It calls for future research to more precisely delineate the indications for surgical intervention in chronic pain syndromes of uncertain etiology and to formulate comprehensive guidelines for addressing rare osteo-muscular conflicts [20,29].

In conclusion, this case report significantly enriches the dialogue surrounding the diagnostic and therapeutic challenges associated with rare musculoskeletal conditions. Advocating for a nuanced approach to the management of chronic pain, it accentuates the importance of advanced diagnostic evaluations and the judicious consideration of surgical options in select instances. The imperative for continued research to refine diagnostic and treatment paradigms is clear, with the ultimate aim of elevating patient care and outcomes within the realm of musculoskeletal medicine [19,29].

The limitations of this case report include its inherent lack of generalizability due to the singular nature of case studies, which prevents the findings from being widely applied to the broader population [30,31]. The absence of long-term follow-up data also restricts our understanding of the durability of the surgical intervention's success, as chronic pain conditions often have variable long-term outcomes. Additionally, the report lacks a comparative analysis with other treatment modalities, limiting the ability to definitively assess the surgery's efficacy against alternative approaches. Lastly, the reliance on advanced imaging for diagnosis points to potential accessibility and cost issues in different healthcare settings, which may limit the applicability of these findings. Despite these limitations, the case provides valuable insights into the management of a rare osteo-muscular conflict, highlighting the need for further research with more extensive follow-up periods to validate the surgical approach discussed.

## Research funding

Authors state no funding involved.

## Author contributions

All authors have accepted responsibility for the entire content of this manuscript and approved its submission.

## Informed consent

Informed consent has been obtained from all individuals included in this study.

## Declaration of competing interest

Authors state no conflict of interest.
